# The After-Care of the Wounded

**Published:** 1915-05-01

**Authors:** W. J. Midelton

**Affiliations:** 112 Charminster Road, Bournemouth.


*To the Editor of* The Hospital.

### The After-Care of the Wounded.

*To the Editor of* The Hospital.

Sir,—I am glad Dr. Ash has called attention to the advantages of counter-irritation boldly and skilfully used. I can speak from many years' experience, and am able to say that this form of treatment fits in well with any other and heightens its efficacy. Even when employed alone its effects are often most gratifying.

One point I persistently call attention to, and that is the advantage that results from the production of a pustular eruption by means of multiple acupuncture followed by the application of a mixture of croton oil, cantharides, and almond oil in definite and carefully regulated proportions. In doing this one is only following Nature's lead. In all rheumatic conditions, especially when not of long standing, the effects are particularly gratifying.—Yours faithfully,

W. J. Midelton.

112 Charminster Road, Bournemouth.

[The letter from Dr. Ash on this subject appeared in our issue of April 17.—Ed. The Hospital.]

